# Effect of core stabilization versus rebound therapy on balance in children with cerebral palsy

**DOI:** 10.1007/s13760-023-02430-8

**Published:** 2024-01-05

**Authors:** Alaa AL-Nemr, Alaa Noureldeen Kora

**Affiliations:** 1 Department of Physical Therapy for Pediatrics, Faculty of Physical Therapy, Cairo University, El‐Tahrir St., Giza, 12613 Egypt; 2 Department of Physical Therapy for Pediatrics and Pediatric Surgery, Faculty of Physical Therapy, Sinai University, East Qantara, Egypt

**Keywords:** Balance, Cerebral palsy, Children, Core stabilization, Hemiplegia, Rebound therapy

## Abstract

**Objective:**

This study aimed to compare the effect of core stabilization exercises and rebound therapy on balance in children with hemiplegic cerebral palsy (CP).

**Methods:**

Fifty- two children of spastic hemiplegic CP aged 5 up to 8 years from both genders were assigned randomly into two groups: core stability and rebound therapy groups. Both groups received 3 sessions/week, 1.5-h training per session, for 12 successive weeks. The measurement was performed at baseline and post-treatment. Balance as a primary outcome for this study was measured by a Biodex Balance System (BBS), and knee extensor strength and functional capacity as secondary outcomes were assessed using a hand-held dynamometer, and a six-minute walk test (6MWT), respectively.

**Results:**

All variables showed a significant improvement after intervention in each group (*p* < 0.0001), with significant improvement in all stability indices (overall, anteroposterior, and mediolateral) in core stability group when compared to rebound therapy group.

**Conclusion:**

Core stability exercises and rebound therapy are recommended in the rehabilitation of children with hemiplegic CP. Core stability exercises were more effective than rebound therapy for balance improvement.

**Trial registration number:**

NCT05739396.

## Introduction

Cerebral palsy (CP) is defined as a collection of movements and postural dysfunctions resulting in restrictions in functional abilities [1]. The lesion in the immature brain in children with CP leads to improper nerve signals to the musculoskeletal system, causing muscular tone abnormality and a decrease in the control of body movements. This results in a decrease in balance, muscle weakness, and disturbance of functional capacity [2].The prevalence of CP is about 1.5 to 4/1000 live births [3].

Balance is the capacity to keep and maintain equilibrium. It is classified into static and dynamic balance [4]. CP gives rise to disturbances in balance, motor functions, activity, and participation.

The production of movement requires stability of the spine and relies on the core muscles to attain sufficient power, endurance, and strength. The core muscles can be clarified as a muscular box with the diaphragm as the roof of the muscular box, the hip girdle and pelvic floor muscles as the bottom, the abdominals in the front, and gluteal and paraspinal muscles in the back [5]. Muscles of this box assist in controlling the movement and position of the central body portion and also provide the basis for extremities movements. Strengthening and core stabilization programs can be done for children with CP to improve both balance and stability [6].

Rebound therapy is the therapeutic use of rebound and rebound-like devices to provide the opportunity for subjects with functional limitations to do recreational activities [7]. It was suggested that rebound therapy help in improving balance, gross motor abilities, and coordination in addition to enjoyment and satisfaction during the treatment sessions [8]. Multiple studies suggested using rebound therapy as a rehabilitation modality for children with CP to increase strength, balance, and functional mobility [7, 8]. The elastic tension that is produced by the springs forms an unstable surface. The movements on unstable surfaces offer an active base upon this movement. Also, the changeable surface can be organized to enhance symmetrical weight bearing, leading to balance improvement [9]. Germain and his colleagues added that rebound therapy enhances the strength of lower limbs, improves functional strength, motor performance, and balance with a high degree of performance with no adverse effects in children with CP [8].

To the extent of our awareness, until now there is no study comparing the effect of core stabilization and rebound therapy on balance in children with CP. The current study aimed to compare the effect of core stabilization and rebound therapy on dynamic standing balance in children with hemiplegic CP. It was hypothesized that there is no difference between the effect of core stabilization compared to rebound therapy on dynamic standing balance in children with hemiplegic CP [10].

## Methods

### Participants

Fifty-two children with hemiplegic CP from the Faculty of Physical Therapy, Cairo University (25 boys, 27 girls) enrolled in this study and complied with the following inclusion criteria: can follow instructions given to them, their chronological age ranged from 5 up to 8 years, muscle tone grade of their lower limbs ranged between 1+ and 2 in the Modified Ashworth scale (MAS), and they were classified as Level II & III of gross motor function in Gross Motor Function Classification System Expanded and Revised (GMFCS E&R). Children were excluded in case of significant problems in vision or hearing, botulinum toxin injection or orthopedic surgery 6 months before the study in the lower limbs, children suffering from epilepsy, osteoporosis, contracture, deformity and/or tightness in upper or lower limbs, recent fracture, vertigo or dizziness.

### Study design

**Fig. 1:**
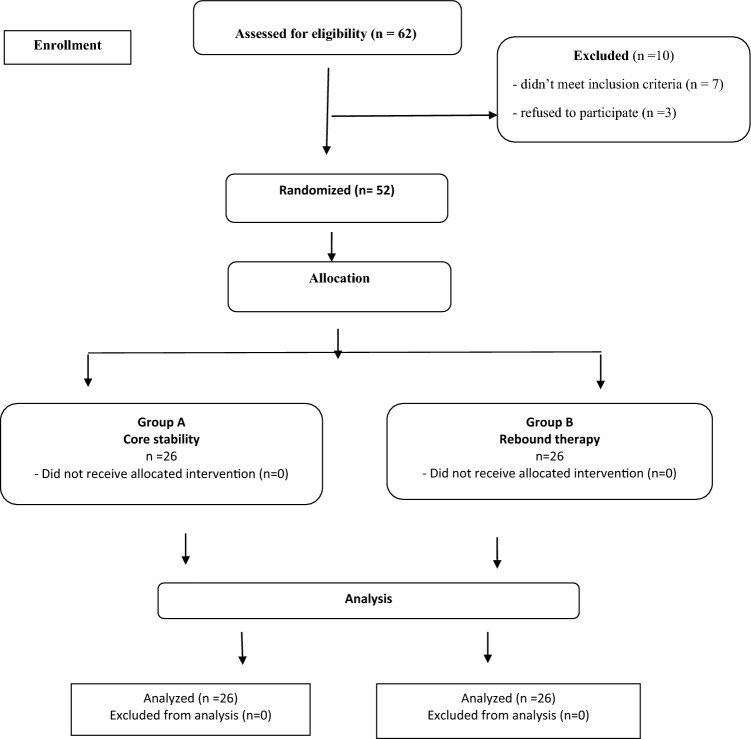
Participants’ flow chart

The current study is a comparative, single-blinded randomized controlled study design, performed between November 2022 and May 2023. Children with hemiplegic CP were randomized by a simple random sampling system into two groups; core stability and rebound therapy using closed sealed envelopes. Recruitment for this study included sixty-two children with hemiplegic CP; parents of three children refused their study participation and seven children did not meet the inclusion criteria. The Consolidated Standards of Reporting Trials (CONSORT) diagram (Fig. 1) displays the study design.

### Intervention

Interventions were described according to the TIDieR checklist, Tables 1 and 2. A designed physical therapy program in conjunction with core stability exercises was applied to one group, Tables 1 and 3, while the other group applied the same designed physical therapy program in conjunction with rebound therapy, Tables 2 and 4.

**Table 1:** Core stability intervention group exercise program according to TIDieR criteria

TIDieR criteria	Core stability intervention group
Name	Core stability Exercises in addition to a Specially designed program of physical therapy
Why	To enhance balance, postural control, stability, muscle strength, and functional capacity
What	Subjects received a 1.5-h physical therapy session The session included (1) neurodevelopmental techniques, (2) proprioceptive training, (3) facilitation of normal movement patterns and inhibition of abnormal ones, (4) facilitation of postural reactions, (5) facilitation of postural mechanism, (6) stretching exercises for shortened muscles, (7) gait training in an open environment, in addition to core stability exercise program with two minutes of rest between exercises including seven exercises including abdominal draw-in, roll-up, superman, hands and knees superman, bridging, crab walk, and plank exercises
Who provided it	12 years experienced pediatric physical therapist
How	A face-to-face, one-to-one session. One pediatric physical therapist per subject
Where	In a quiet environment without any sort of disturbance in the Pediatric Outpatient Clinic in the Faculty of Physical Therapy Cairo University
When and how much	Duration: 12 consecutive weeks Frequency: 3 times/week No. of sessions: 36 sessions
Tailoring	The specified details of balance, muscle strength and functional capacity were modulated for each subject individually according to his baseline abilities
Modifications	No modifications were made during the intervention
How well	A blinded Physical Therapist evaluated the subjects initially at baseline and after the intervention. Records of balance, postural control, stability, muscle strength & functional capacity were explained to the caregivers

**Table 2:** Rebound therapy intervention group exercise program according to TIDieR criteria

TIDieR criteria	Rebound Therapy Intervention Group
Name	Rebound Therapy Exercises in addition to a Specially designed program of physical therapy
Why	To enhance balance, stability, strength, and functional capacity
What	Subjects received a 1.5-h physical therapy session The session included (1) neurodevelopmental techniques, (2) proprioceptive training, (3) facilitation of normal movement patterns and inhibition of abnormal ones, (4) facilitation of postural reactions, (5) facilitation of postural mechanism, (6) stretching exercises for shortened muscles, (7) gait training in an open environment, in addition to rebound therapy exercise program with two minutes of rest between exercises including active bounce, squatting exercises, catching and throwing a ball, and jumping
Who provided it	12 years experienced pediatric physical therapist
How	A face-to-face, one-to-one session. One pediatric physical therapist per subject
Where	In a quiet environment without any sort of disturbance in the Pediatric Outpatient Clinic in the Faculty of Physical Therapy Cairo University
When and how much	Duration: 12 consecutive weeks Frequency: 3 times/week No. of Sessions: 36 sessions
Tailoring	The specified details of balance, strength and functional capacity were modulated for each subject individually according to his baseline abilities
Modifications	No modifications were made during the intervention
How well	A blinded Physical Therapist evaluated the subjects initially at baseline and after the intervention. Records of balance, postural control, stability, muscle strength and functional capacity were explained to the caregivers

**Table 3:** Core stability prescribed exercises

Exercise	Description
Abdominal draw in	From supine, prone and squat position
Roll up	Lifting both legs to the chest while lying on the back and holding both knees with both arms. Gradually, a ball was thrown to the child and he/she was asked to kick it up back with both feet
Superman	lifting both arms and head up while lying on abdomen with both arms in front of the child and legs straight, then lifting both legs and gradually lifting arms and legs at the same time
Hands and knees superman	Raising one arm and the opposite leg straight from quadruped position. Then raising the other side
Bridging	Raising the bottom up while lying on the back with knees bent and feet on ground. Then, gradually holding a ball between both knees while bridging to prevent flopping of knees outward. Finally, lifting one leg up with straight knee while bridging
Crab walk	Lifting the bottom up with arms extended behind the child and feet on the floor with flexed knees, then gradually walk while in this position
Plank	Pushing up from prone on both hands and toes then gradually touching one of the child’s hands by the therapist’s hands

**Table 4:** Rebound therapy prescribed exercises

Exercise	Description
Active bounce	bouncing lonely on a mini-trampoline
Squatting exercises	Bending both knees with arms alongside the body until both thighs are parallel to the floor while keeping feet shoulder-width apart and then gradually keeping feet together
Catching and throwing a ball	Holding a large ball on mini-trampoline in both hands, then throwing the ball up to the therapist and catching it while keeping feet shoulder-width apart, then the same exercise while keeping both feet together from various positions including kneeling, half kneeling and standing
Jumping	While standing on mini- trampoline, jumping forward, sideways, and backward with eyes opened

### Measurements

Biodex balance system (BBS) was used as a measurement for dynamic standing balance which was considered the primary outcome of this study, while a hand-held dynamometer and a 6-min walk test (6MWT) were used as secondary outcome measures for assessing knee extensor strength and functional capacity respectively.

The BBS is a sophisticated reliable and objective assessment device for balance (BBS; Biodex Medical Systems, Inc, Shirley, NY). It has a platform that can shift in medial, lateral, anterior, and posterior directions to assess the overall stability index (OASI), mediolateral stability index (MLSI), and anteroposterior stability index (APSI) [11]. Instructions have been given to all children about how to perform the test before starting the evaluation. According to the child’s height, handrails had to be adjusted to obtain safety for all children while using the device but they were not allowed to use them during testing. Standing on a platform barefoot with arms beside the body and looking forward at an adjustable display screen for visual biofeedback was required. The Dynamic Balance operation was chosen as a method of assessment and the child was required to keep the cursor at the middle of the screen during the platform movement. BBS has eight stability levels; level 1 represents the level with the least stability and level 8 represents the level with the most stability. In this study, the measurements were conducted at level 6 because it was suitable for all participating children [12, 13]. Three trials were performed for each child, each one lasted 30 s and the mean value was used while analyzing the data. Higher scores indicated poorer balance.

A handheld dynamometer is an objective, valid and reliable instrument in children with CP for measuring isometric muscle strength (Nicholas Manual Muscle Test system, Model 01163; Lafayette Instrument Company, Lafayette, IN) [14]. It was selected in this study for measuring the affected knee extensors strength. It was set to read force in pounds. From sitting position with knees and hips flexed 90° while stabilizing the pelvis and the other non-tested limb, all children were instructed to perform maximal isometric contraction against dynamometer resistance which was placed at the anterior side of the distal tibia, 10 cm above the lateral malleolus [15]. Three attempts were recorded. The application of the first attempt was for familiarization, and the second and third attempts were averaged to obtain the score. One minute rest between trials was allowed to avoid possible fatigue.

Six-minute walk test is a submaximal, valid, and reliable test that is used to evaluate endurance and aerobic capacity in children with CP [16]. Evaluation procedures occurred in a straight corridor with a flat hard surface of 30 m long, without any obstacles. Children were asked to wear comfortable clothing. During the test procedure, verbal instructions and encouragement were given to all participants. Children were asked to walk as far as they could for a period of 6 min. After ending the test, the distance that the children covered was recorded in meters [17].

### Statistical analysis

Calculation of the sample size prior to the study was done using the statistical software “G*POWER” (version 3.1.9.4; Franz Faul, Universitat Kiel, Germany) using large effect size, *β* = 0.2, *α* = 0.05. The detected sample size for our study was *N* = 52; with 26 children for each group. 62 children were recruited for possible dropouts.

The statistical package for social studies (SPSS) version 25 for Windows (IBM SPSS, Chicago, IL, USA) was selected to conduct the statistical analysis. The *t*-test was selected to analyze the characteristics of subjects between groups. Chi-square test was selected to analyze the comparison of affected side distribution and sex between groups. The Shapiro–Wilk test was selected for checking if the data are normally distributed, and Levene’s test was selected for testing between groups homogeneity. Mixed repeated measures MANOVA was used for comparing within and between treatment effects for all outcomes. For subsequent multiple comparisons, multiple tests were performed using the Bonferroni correction. “*p* < 0.05” was used to set the level of significance for all statistical tests.

## Results

The subject characteristics are shown in Table 5. Age, height, weight, the distribution of sex, and affected side showed no significant difference between groups (*p* > 0.05).

**Table 5:** Comparison of participants’ baseline characteristics between core stability and rebound therapy groups

	Groups				Test statistics
	Core stability group		Rebound therapy group		
	*x̄* ± SD	*n* (%)	*x̄* ± SD	*n* (%)	*p* value
Age (years)	8.52 ± 0.67		8.20 ± 0.66		0.09^a^
Height (cm)	117 ± 2.3		118 ± 2.6		0.65^a^
Weight (kg)	29.4 ± 3.4		29.2 ± 3.1		0.82^a^
Boys/girls		12/14 (46%/54%)		13/13 (50%/50%)	0.52^b^
Affected side, right/left		14/12 (54%/46%)		15/11 (58%/42%)	0.11^b^
GMFCS E&R					
Level II		10 (38.5%)		11 (42.3%)	
Level III		16 (61.5%)		15 (57.7%)	
Muscle tone grade					
1+		9 (34.6%) 17		8(30.8%)	
2		(65.4%)		18 (69.2%)	

*x̄* mean, *SD* standard deviation, *n* number, *%* percentage, *a* *t*-test, *b* Chi-square test

### Effect of treatment on stability indices, knee extensors strength, and 6MWT

A significant interaction of treatment and time was detected as revealed by Mixed MANOVA (*η*^2^ = 0.38; *F* (5,46) = 5.7, *p* = 0.0001). A significant main effect of treatment was detected (*η*^2^ = 0.393; *F* (5,46) = 5.96, *p* = 0.0001). A significant main effect of time was detected (*η*^2^ = 0.98; *F* (5,46) = 756.2, *p* = 0.0001).

### Comparison within the group

A decrease in all stability indices was significantly detected post-treatment in both groups, when compared to pre-treatment values (*p* < 0.0001). In addition, an increase in knee extensor strength and 6MWT was significantly detected post-treatment when compared to pre-treatment values (*p* < 0.0001) in both groups as shown in Table 6.

**Table 6:** Changes in stability indices, knee extensors strength, and 6MWT within and between groups

	Pre-treatment			Post-treatment				
	Core stability group	Rebound therapy group	*p* value	Core stability group	Rebound therapy group	*p* value	Pre-VS post	Pre-VS post
							Core stability group	Rebound therapy group
	*x̄* ± SD	*x̄* ± SD		*x̄* ± SD	*x̄* ± SD		*p* value	*p* value
Stability index								
Anteroposterior	2.9 ± 0.2	2.8 ± 0.3	0.343	1.4 ± 0.2	1.6 ± 0.1	0.0001*	0.0001*	0.0001*
Mediolateral	2.6 ± 0.1	2.7 ± 0.2	0.109	1.1 ± 0.1	1.4 ± 0.2	0.0001*	0.0001*	0.0001*
Overall	3.1 ± 0.2	3.1 ± 0.3	0.349	1.6 ± 0.2	1.9 ± 0.5	0.0001*	0.0001*	0.0001*
Knee extensors strength	14.1 ± 1.1	13.9 ± 1.2	0.671	16.1 ± 1.2	15.5 ± 1.3	0.103	0.0001*	0.0001*
6 MWT (m)	283.3 ± 7.1	282.1 ± 7.3	0.566	300.6 ± 7.9	296.4 ± 5.2	0.066	0.0001*	0.0001*

*x̄* mean, *SD* standard deviation, *6MWT* 6 min walk test, *m* minute, * significant

### Comparison between groups

**Fig. 2:**
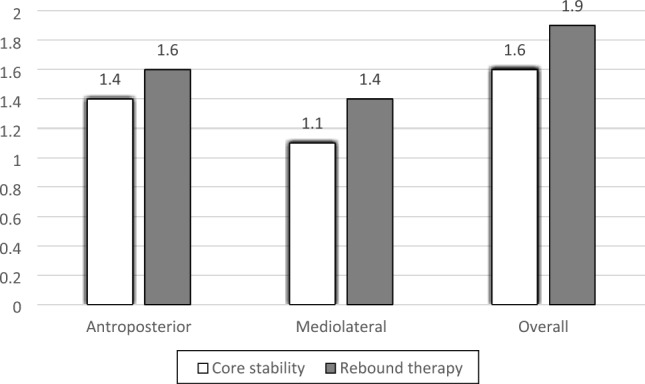
Comparison of post-treatment mean values of different balance outcomes between groups

**Fig. 3:**
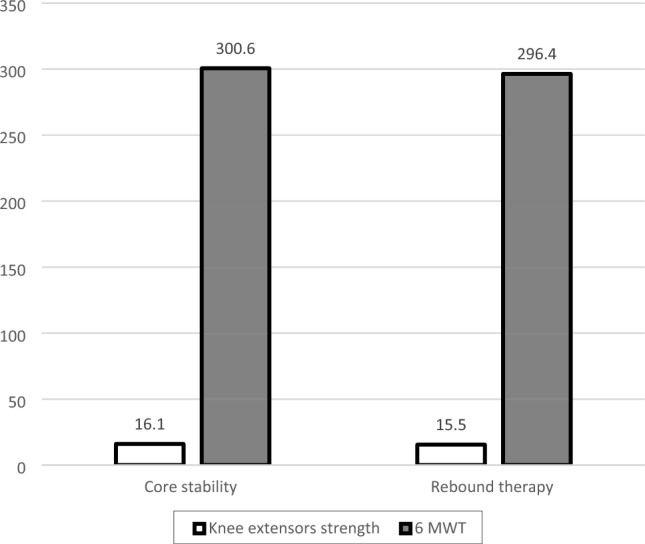
Comparison of post-treatment mean values of knee extensors strength and 6 MWT between groups

Comparison between groups is shown in Table 6. No significant difference was observed between core stabilization and rebound therapy groups in all outcome measures pre-treatment (*p* > 0.05). A significant decrease in all stability indices of the core stability group compared with that of the rebound therapy group post-treatment was detected (*p* < 0.0001) as shown in Fig. 2, while there was a non-significant difference in the strength of knee extensors and 6MWT when comparing the two groups post-treatment (*p* > 0.05) as shown in Fig. 3.

## Discussion

The objective of the current study was to compare the effect of core stabilization exercises and rebound therapy on balance in children with hemiplegic CP. The hypothesis was rejected and there was a significant difference between core stability exercises and rebound therapy in favor of core stability.

Maclennan and coworkers defined CP as a heterogeneous condition. Since there are numerous causative pathways and different types and degrees of dysfunction within the CP clinical spectrum, CP might be better described as "the cerebral palsies." [18]. The neurological subtype of CP is a powerful predictor of functional status in children with CP [19]. Children with hemiplegic type of CP typically exhibit higher gross motor function scores. As a result, they have less activity restrictions than the other types. [20]. Specifying type of CP was shown in different balance studies for homogeneity of the cases [21–23]. In addition, diplegic patients exhibited weaker postural balance compared with hemiplegic CP patients in other studies [24, 25]. For these reasons, only spastic hemiplegic CP children with levels II &III of GMFCS were selected to be included in our study.

A significant improvement of both groups in all types of stability indices was shown post-treatment in comparison with their pre-treatment results. The core stability group’s stability indices results showed significantly better dynamic balance than the rebound therapy group**.**

The core stability group in our study showed a significant improvement in balance post-treatment in comparison with pre-treatment results. The main reason for this improvement might be related to improving the trunk muscle strength after core stability training programs as core stabilization improve the strength and the control of the trunk's stabilizing muscles which is directly linked to balance in children with spastic CP [26]. Core stability training activates the trunk musculature by proprioceptive stimulation. In addition, the somatosensory control of the vestibular system is also stimulated during the core stability exercise [27]. Improvement of balance in the core stability group in our study has been supported by the findings of the study by Rana and his colleagues which concluded that the stability of the trunk is a significant core element of coordination and balance in children with CP [28]. Our findings are also supported by previous studies which studied trunk stability exercises and their effect on children’s balance in different disabilities including diplegic and hemiplegic children with CP [29], children with ataxic CP [27], children with Down syndrome [30], and also, in normal children [31].

The trunk muscles activities are clearly correlated with the lower extremity function and movement. The core stability program can improve the postural activity of the extremities by stimulating the feed-forward system [32]. Thus, improving both lower limbs’ muscle strength and their functional activities which can justify the improvement in the strength of knee extensors and functional capacity in the core stability group after core stability exercises. These results are consistent with Hoppes and his colleagues who stated that 8 weeks of a core stabilization program can improve endurance [33]. Core stability problems were identified as potential risk factors for injuries of the lower extremity [32]. Güngör and his colleagues stated that both home and supervised Pilates-based core stability exercises could improve postural sway and lower extremity muscle strength in patients with multiple sclerosis [34].

Rebound therapy creates situations that improve both static and dynamic balance mechanisms via vestibular and proprioceptive stimulation that enhances balance and postural stability and decreases the risk of falling [35]. It was proven to improve hip moment generation resulting in increasing dynamic stability [36] which can illustrate the significant balance improvement which is detected in the rebound therapy group.

The current study results were supported by Abd-Elmonem and Elhady results who studied the rebound therapy effect on balance in 40 children with diplegic CP whose balance was evaluated using the dynamic test in BBS. Their study revealed a significant improvement in balance after the rebound therapy exercise program [37]. Our results are also supported by previous studies, which examined the rebound therapy effect on balance in children with different disabilities including autism spectrum disorder [38], and Down syndrome [39].

Skin, muscle, and joint receptors as well as the vestibular system can be stimulated by rebound exercises, resulting in muscle co-contraction, modulation of muscle tone, and increase the knee extensor moments leading to an increase in knee joint stability [40]. The current study revealed that the strength of knee extensors and functional capacity were significantly improved after rebound therapy. These results may be based on the biomechanical effect of rebound therapy on joint range of motion and stretch reflex [39].

Our results came in agreement with a study by Azab and his colleagues who reported that trampoline-based stretch–shortening cycle exercises have distinct effects on muscle strength in children with Down syndrome [39]. Claesson and his colleagues also conducted a study that revealed the effect of somatosensory training using rebound therapy and showed a significant improvement in balance and functional capacity using Berg Balance Scale and 10-min walk test respectively [41].

The current study has many limitations; only one type of CP (hemiplegic type), specific age group (from 5 up to 8 years), and two levels of GMFCS (level II and III) were allowed to be included in this study. So, additional studies are required to be performed on children with different levels of GMFCS, different ages, and other types of CP. Also, absence of follow-up for participants. So, future studies are recommended to detect and illustrate the maintenance of the improvement gained.

## Conclusion

In this study, a significant dynamic standing balance improvement of the core stability group when compared to the rebound therapy group was revealed when comparing the two intervention groups after 12 weeks intervention. The current study concluded that including both core stability and rebound therapy during the rehabilitation of children with hemiplegic CP is helpful when the treatment plan aims to improve their balance. However, core stabilization produces significantly more favorable effects on balance than rebound therapy when added to a designed physical therapy program in those children.

## References

[1] Cadwgan J, Goodwin J, Fairhurst C (2019). Fifteen-minute consultation: modern-day art and science of managing cerebral palsy. Arch Dis Child Educ Pract.

[2] Patel DR, Neelakantan M, Pandher K, Merrick J (2020). Cerebral palsy in children: a clinical overview. Transl Pediatr.

[3] Stavsky M, Mor O, Mastrolia SA, Greenbaum S, Than NG, Erez O (2017). Cerebral palsy-trends in epidemiology and recent development in prenatal mechanisms of disease, treatment, and prevention. Front Pediatr.

[4] Marchesi G, De Luca A, Squeri V, De Michieli L, Vallone F, Pilotto A, Leo A, Casadio M, Canessa A (2022). A lifespan approach to balance in static and dynamic conditions: the effect of age on balance abilities. Front Neurol.

[5] Akuthota V, Ferreiro A, Moore T, Fredericson M (2008). Core stability exercise principles. Curr Sports Med Rep.

[6] Dodd KJ, Taylor NF, Damiano DL (2002). A systematic review of the effectiveness of strength-training programs for people with cerebral palsy. Arch Phys Med Rehabil.

[7] Kora AN, Abdelaziem FH (2020). The effect of rebound therapy on gross motor functions in a child with spastic cerebral palsy: a case study. BioSci Rev.

[8] Germain AM, Blackmore AM, Gibson N, Newell B, Williams SA (2019). Effects of adaptive bungee trampolining for children with cerebral palsy: a single-subject study. Pediatr Phys Ther.

[9] Behm DG, Muehlbauer T, Kibele A, Granacher U (2015). Effects of strength training using unstable surfaces on strength, power and balance performance across the lifespan: a systematic review and meta-analysis. Sports Med.

[10] Boruch R (2007). The null hypothesis is not called that for nothing: Statistical tests in randomized trials. J Exp Criminol.

[11] Arifin N, Abu Osman NA, Wan Abas WAB (2014). Intrarater test-retest reliability of static and dynamic stability indexes measurement using the Biodex Stability System during unilateral stance. J Appl Biomech.

[12] Emara H, ElGohary T, Khaled O (2016). Using biodex balance training system to improve postural control in spastic diplegic cerebral palsy children: a biomechanical perspective. Int J Ther Rehabilit Res.

[13] El-gohary TM, Emara HA, Al-Shenqiti A, Hegazy FA (2017). Biodex balance training versus conventional balance training for children with spastic diplegia. J Taibah Univ Med Sci.

[14] Crompton J, Galea MP, Phillips B (2007). Hand-held dynamometry for muscle strength measurement in children with cerebral palsy. Dev Med Child Neurol.

[15] Hirunyaphinun B, Taweetanalarp S, Tantisuwat A (2019). Relationships between lower extremity strength and the multi-directional reach test in children aged 7 to 12 years. Hong Kong Physiother J.

[16] Nsenga Leunkeu A, Shephard RJ, Ahmaidi S (2012). Six-minute walk test in children with cerebral palsy gross motor function classification system levels I and II: reproducibility, validity, and training effects. Arch Phys Med Rehabil.

[17] Agarwala P, Salzman SH (2020). Six-minute walk test clinical role, technique, coding, and reimbursement. Chest.

[18] MacLennan AH, Thompson SC, Gecz J (2015). Cerebral palsy: causes, pathways, and the role of genetic variants. Am J Obstet Gynecol.

[19] Shevell MI, Dagenais L, Hall N (2009). The relationship of cerebral palsy subtype and functional motor impairment: a population-based study. Dev Med Child Neurol.

[20] Palisano R, Rosenbaum P, Walter S, Russell D, Wood E, Galuppi B (1997). Development and reliability of a system to classify gross motor function in children with cerebral palsy. Dev Med Child Neurol.

[21] Abd Allah NE, Kamal HM, Abd El-Nabie WAE-H (2023). Association between pelvic inclination and balance in children with spastic diplegia. Bull Fac Phys Ther.

[22] Kenis-Coskun O, Giray E, Eren B, Ozkok O, Karadag-Saygi E (2016). Evaluation of postural stability in children with hemiplegic cerebral palsy. J Phys Ther Sci.

[23] Mohammed AH, El-Serougy HR, Karim AEA, Sakr M, Sheha SM (2023). Correlation between selective motor control of the lower extremities and balance in spastic hemiplegic cerebral palsy: a randomized controlled trial. BMC Sports Sci Med Rehabil.

[24] Girolami GL, Shiratori T, Aruin AS (2011). Anticipatory postural adjustments in children with hemiplegia and diplegia. J Electromyogr Kinesiol.

[25] Rojas VG, Rebolledo GM, Muñoz EG, Cortés NI, Gaete CB, Delgado CM (2013). Differences in standing balance between patients with diplegic and hemiplegic cerebral palsy. Neural Regen Res.

[26] Panibatla S, Kumar V, Narayan A (2017). Relationship between trunk control and balance in children with spastic cerebral palsy: a cross-sectional study. J Clin Diagn Res.

[27] Elshafey MA, Abdrabo MS, Elnaggar RK (2022). Effects of a core stability exercise program on balance and coordination in children with cerebellar ataxic cerebral palsy. J Musculoskelet Neuronal Interact.

[28] Mehmood Rana F, Jabbar S, Khalid M, Umar S, Ali M, Batool U (2022). Effect of trunk stabilization exercises on static and dynamic sitting balance among children with cerebral palsy: a randomized control trial. Pak J Med Health Sci.

[29] Ali MS, Awad AS, Elassal MI (2019). The effect of two therapeutic interventions on balance in children with spastic cerebral palsy: a comparative study. J Taibah Univ Med Sci.

[30] Alsakhawi RS, Elshafey MA (2019). Effect of core stability exercises and treadmill training on balance in children with down syndrome: randomized controlled trial. Adv Ther.

[31] Lengkana AS, Tangkudung J, Asmawi A (2019). The effect of core stability exercise (CSE) on balance in primary school students. J Educ Health Sport.

[32] Willson JD, Dougherty CP, Ireland ML, Davis IMC (2005). Core stability and its relationship to lower extremity function and injury. J Am Acad Orthop Surg.

[33] Hoppes CW, Sperier AD, Hopkins CF, Griffiths BD, Principe MF, Schnall BL, Bell JC, Koppenhaver SL (2016). The efficacy of an eight-week core stabilization program on core muscle function and endurance: a randomized trial. Int J Sports Phys Ther.

[34] Güngör F, Tarakci E, Özdemir-Acar Z, Soysal A (2022). The effects of supervised versus home pilates-based core stability training on lower extremity muscle strength and postural sway in people with multiple sclerosis. Mult Scler.

[35] Allum JHJ, Honegger F (1998). Interactions between vestibular and proprioceptive inputs triggering and modulating human balance-correcting responses differ across muscles. Exp Brain Res.

[36] Aragão FA, Karamanidis K, Vaz MA, Arampatzis A (2011). Mini-trampoline exercise related to mechanisms of dynamic stability improves the ability to regain balance in elderly. J Electromyogr Kinesiol.

[37] Abd-Elmonem AM, Abd Elhady HS (2018). Effect of rebound exercises on balance in children with spastic diplegia. Int J Ther Rehabil.

[38] Lourenço C, Esteves D, Lourenço C, Esteves D (2004). Motor intervention and assessment instruments in autism spectrum disorders. Creat Educ.

[39] Azab AR, Mahmoud WS, Basha MA, Hassan SM, Morgan EN, Elsayed AE, Kamel FH, Elnaggar RK (2022). Distinct effects of trampoline-based stretch-shortening cycle exercises on muscle strength and postural control in children with Down syndrome: a randomized controlled study. Eur Rev Med Pharmacol Sci.

[40] Akasaka K, Tamura A, Katsuta A, Sagawa A, Otsudo T, Okubo Y, Sawada Y, Hall T (2017). Does trampoline or hard surface jumping influence lower extremity alignment?. J Phys Ther Sci.

[41] Claesson IM, Ståhle A, Lökk J, Grooten WJA (2018). Somatosensory focused balance training without cues can improve balance and gait in early Parkinson’s disease—a randomised pilot study. Eur J Physiother.

